# Nerve Injury-Induced Immune Responses in the Taste Bud Target Field

**DOI:** 10.3390/ijms27041839

**Published:** 2026-02-14

**Authors:** Josh Brown, Yonggang Bao, Tagwa Ali, Emma Heisey, Osarume Ogala, Taylor Hardeman, Lynnette McCluskey

**Affiliations:** Department of Neuroscience and Regenerative Medicine, Augusta University, Augusta, GA 30912, USA; jbrown65@augusta.edu (J.B.); ybao@augusta.edu (Y.B.); taali@augusta.edu (T.A.); eheisey@augusta.edu (E.H.); oogala@augusta.edu (O.O.); tahardeman@augusta.edu (T.H.)

**Keywords:** chorda tympani nerve, axotomy, degeneration, Interleukin-1, gustatory, macrophages, regeneration

## Abstract

Damage to the chorda tympani (CT) nerve through trauma or experimental nerve axotomy results in the degeneration of anterior taste buds and taste loss. Our previous work demonstrated that interleukin-1 receptor 1 (*Il1r*) signaling is required for taste bud regeneration and the recovery of taste function. However, the effects of experimental axotomy on immune responses in the absence of *Il1r* signaling remain unclear. Here we performed unilateral CT sectioning in *Il1r* KO or wild-type mice to observe changes in innate immune cell populations in the anterior taste field. We found that CD45+ immune cells, CD68+ and CD206+ M2-like macrophages are significantly increased near anterior taste buds at days two and five post-injury in wild-type but not *Il1r* KO mice. However, taste buds degenerated at similar time points in both strains, suggesting that a suppressed immune responses in the absence of *Il1r* signaling is not the primary reason for later functional deficits. The presence of pro-regenerative M2-like macrophages may play a role in later taste bud regeneration and functional recovery in the injured peripheral taste system.

## 1. Introduction

Taste buds are heterogenous chemosensory organs that transmit information about chemical stimuli from the oral cavity to the gustatory cortex. Similarly to epithelial cells, taste bud cells are not permanent and have a turnover rate of 7–30 days, with each taste bud containing 40–100 specialized taste cells. Taste buds are composed of four main taste receptor cell types. Type I cells are “glial-like” with processes that encompass other cell types in the taste bud. Type II and type III cells are directly responsible for the transduction of taste information, with type II responding to bitter, sweet or umami stimuli and type III responding to sour and salty tastants. Post-mitotic type IV cells at the base of the taste bud differentiate into the other three cell types [1,2].

Taste buds are innervated by and receive trophic support from sensory afferent nerve fibers, as first reported 150 years ago [3]. Anterior taste buds in fungiform papillae are innervated ipsilaterally by one of two chorda tympani (CT) nerves on each side of the tongue. Unlike neurons in the central nervous system, peripheral gustatory axons have the ability to regenerate after injury. In adult rodents and other mammals, experimental axotomy of the chorda tympani nerve is followed by degeneration of distal axons associated with taste buds [1,4,5,6]. However, within 2–4 four weeks in gerbils and mice, respectively, CT fibers and taste buds regenerate and normal responses to tastants are restored [7,8,9,10]. Taste bud regeneration ultimately culminates in behavioral as well as neurophysiological recovery [11]. The sense of taste is vulnerable to damage by chemotherapeutic treatment, infection, trauma, or through direct damage to the CT nerve during surgical or dental procedures [6,12,13,14,15,16]. Lasting deficits in taste perception remain in some patients, highlighting the need for a better understanding of mechanisms mediating the recovery of gustatory function [17]. The plasticity of the peripheral taste system and bilateral innervation of anterior taste buds make the rodent CT injury model ideal for elucidating mechanisms of recovery [6].

Gustatory nerve injury and taste bud degeneration elicits immune responses and the activation of inflammatory pathways (reviewed in [6]). Leukocytes identified by morphology and ultrastructure have been observed in the degenerating taste fields of dog, rat, and mouse nerve transection models [4,5,6,18]. Less is known about the specific identity and dynamics of immune cell responses to taste bud denervation in wild-type rodents. Our group demonstrated a rapid influx of neutrophils in the degenerating taste bud field within 6 h followed by macrophage responses over the next 48 h [19,20,21,22,23,24].

Nerve injury also upregulates chemokine, adhesion molecule, and cytokine responses in the anterior taste field [24,25,26,27,28]. The IL-1 receptor (IL-1R)1 and ligands IL-1α and IL-1β are expressed in taste buds, axons, and immune cells in response to axotomy [28,29]. IL-1α and IL-1β bind to IL-1R to trigger intracellular signaling and activate inflammatory responses. In the absence of *Il1r*, these responses are typically blunted in other tissues, especially in the context of infection or injury [30,31,32,33]. However, the lingual mRNA levels of the pro-inflammatory cytokine *Il1b* are upregulated at day 56-60 in *Il1r* KO compared to wild-type C57BL/6J mice, which could induce inflammation at this late period [7]. This dysregulation, combined with the pleiotropism and redundancy of cytokine pathways, highlights the need to investigate immune responses in the taste bud target field after CT nerve injury.

*Il1r* KO mice receiving CT axotomy exhibit altered cytokine response dynamics in the injured taste bud field, dysregulated taste progenitor cell proliferation, and incomplete taste bud regeneration. Importantly, these changes result in absent or minimal neural responses to taste stimuli even at >14 weeks post-injury, compared to the recovery of normal taste function at 4 weeks in wild-type mice [7]. These results support the requirement for IL-1R signaling in taste bud regeneration and functional recovery. However, it is unknown which specific immune cell populations might be altered in the denervated taste bud target field in *Il1r* KO mice that do not recover taste function. Here, we quantified changes in immune responses in the absence of IL-1R signaling. We also used M1 proinflammatory and M2 pro-regenerative macrophage activation markers to identify subtype specific responses, in contrast to our previous studies in rats using a general macrophage marker [24,34,35]. To accomplish this, we performed surgical CT axotomy on wild-type (wild-type) and *Il1r* KO mice [29] then measured their innate immune cell responses [4,5,18] at different times post-injury. We also tested whether dysregulated immune responses in *Il1r* KO mice delay taste bud degeneration at day 10, potentially preventing later, efficient taste cell regeneration. Identifying the dynamics of specific immune responses will potentially allow for more targeted therapeutic treatments in the restoration of gustatory function after nerve injury, chemotherapy, or infection.

## 2. Results

### 2.1. CD45+ Immune Cells Rapidly Infiltrate Axotomized Taste Papillae in Wild-Type but Not Il1r KO Mice

**Figure 1:**
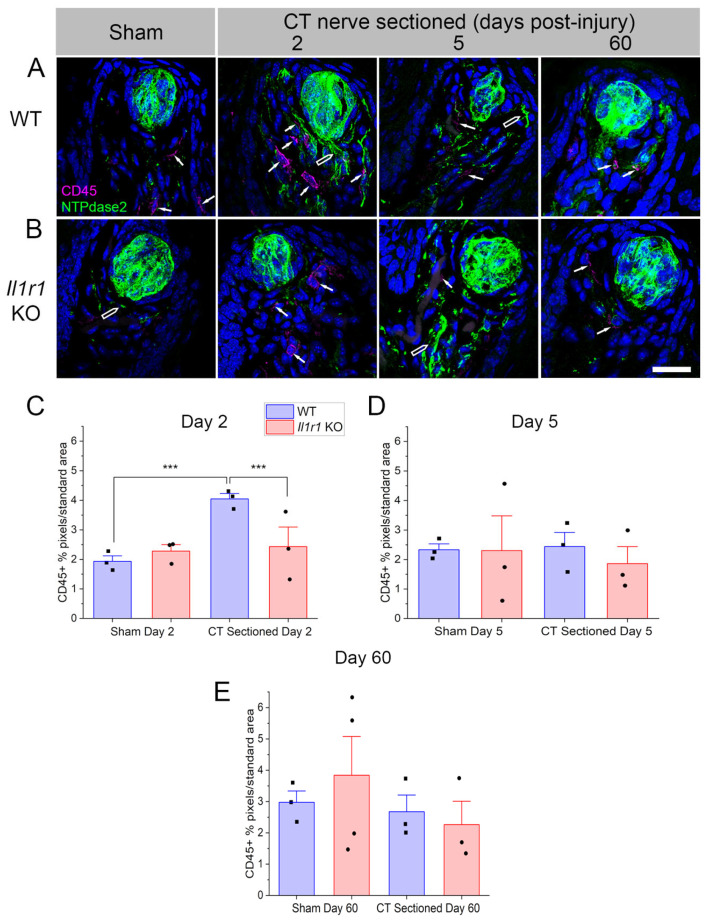
CD45+ immune responses are suppressed in the early post-injury period in *Il1r* KO compared to wild-type mice. (**A**) The density of CD45+ immune cell responses (magenta, white arrows) increases near denervated taste buds at day 2 post-injury in wild-type mice. Taste buds are visualized with the type I taste cell marker NTPdase2 (green), which also labels nerve fibers in the papilla (open arrows). DAPI (blue) was used to identify cell nuclei. CD45+ immune responses return to baseline at day 5 post-injury and remain at low levels at day 60 in wild-type papillae with regenerated, functional taste buds. (**B**) In *Il1r* KO mice, few CD45+ immune cells occupy taste papillae at any post-injury period tested. (**C**) CD45+ pixels were thresholded, expressed as the percent/standard area, and summed at each period (*n* = 3–4 mice/group). There was a significant increase in the mean (+SEM) CD45+ response at day 2 post-injury compared to sham wild-type mice or axotomized *Il1r* KO mice. Bonferroni post-tests revealed no significant differences in CD45+ immune responses among groups at (**D**) day 5 or (**E**) day 60. *** *p* < 0.0001. Scale bar in (**B**) = 20 µm.

CD45+, a pan-leukocyte cell marker, labeled cells in fungiform papillae, the lamina propria immediately beneath the lingual epithelium, and throughout the deeper muscle layer of the anterior tongue. We are interested in immune cells that potentially affect taste bud integrity and function, so we spatially limited our analyses to fungiform papillae and nearby lamina propria. CD45+ immune cells were rarely observed within the lingual epithelium or inside taste bud borders in sham-sectioned mice. In response to nerve sectioning, immune cells infiltrated taste papillae and took on an activated morphology with projections extending from the cell (Figure 1A, white arrows). In contrast, CD45+ cells observed in the lingual muscle typically had a more rounded morphology (Figure 1B). NTPdase2, which labels the numerous type I glial-like taste cells, was used to identify taste buds. We note that NTPdase2 expression is also present in lingual nerve fibers as previously reported [36] (Figure 1A, open arrows).

We analyzed immune responses at day 2, day 5, and day 60 post-injury. The timing was based on previous studies in rats demonstrating elevated macrophage responses during the first week after CT nerve sectioning. At day 60 post-axotomy, wild-type C57BL/6J mice had recovered normal taste function for over 5 weeks, while *Il1r* KO mice still exhibited minimal taste responsiveness [7]. We hypothesized that inappropriate immune responses at this later period could underlie the absence of taste bud regeneration and functional recovery in KO mice. The statistics are reported in Table 1.

Given the wispy processes of CD45+ immune cells, we thresholded immunopositive pixels in standard-sized regions of interest as in previous studies [7,24,29]. These analyses revealed early, dramatic differences in CD45+ immune responses to axotomy in wild-type and *Il1r* KO groups (Figure 1C). At day 2 post-injury, there were significant main effects of surgical treatment (*p* = 0.0006), mouse strain (*p* = 0.0191), and their interaction (*p* = 0.0006) on immune cell density in taste cell papillae and lamina propria. Specifically, lingual immune cells were significantly elevated in CT-sectioned compared to sham-sectioned control mice (*p* = 0.0001). Notably, CD45+ cell density increased in wild-type compared to *Il1r* KO axotomized mice (Figure 1A,B; *p* = 0.0010), as immune responses remained at baseline sham levels in *Il1r* KO mice. As shown in Figure 1D, at day 5 post-axotomy, there was a significant main effect of mouse strain (*p* = 0.0473), but no difference between groups in post-tests. At day 60 post-injury (Figure 1A,D), despite variability in the sham *Il1r* KO group, there was a significant effect of surgical treatment on CD45+ cell density (*p* = 0.0188) that was not reflected in post-tests. These results demonstrated that CD45+ leukocyte responses are elevated in wild-type but not *Il1r* KO mice at day 2 post-nerve injury.

### 2.2. CD68+ Macrophage Density Increases in Wild-Type Compared to Il1r KO Mouse Taste Papillae

**Figure 2:**
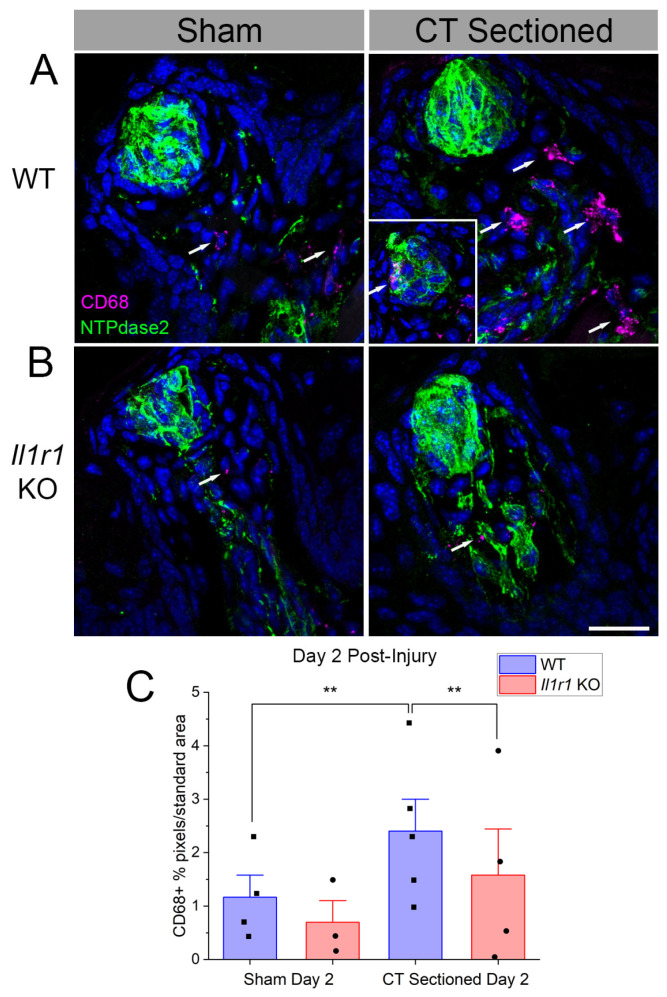
CD68+ macrophage density increases at day 2 post-sectioning in wild-type but not *Il1r* KO mice. (**A**) Many CD68+ macrophages (magenta, white arrows) are present in wild-type taste papillae at day 2 after axotomy compared to sham controls. Occasionally, macrophages infiltrated denervated taste buds (inset on right). (**B**) Few macrophages were observed in *Il1r* KO papillae even after axotomy. (**C**) Quantification of CD68+ pixels/standard area demonstrates a significant increase in the mean (+SEM) level of macrophage response at day 2 post-injury in wild-type mice. DAPI (blue) was used to identify cell nuclei *n* = 3–4 mice/group. ** *p* < 0.001. Scale bar in (**B**) = 20 µm.

We tested whether CD68+ macrophages contribute to the CD45+ leukocyte responses. Based on the results of the CD45 analysis, we focused on day 2 post-injury since immune responses returned to baseline across all mice strains and treatments at day 5 and 60 post-injury. The identified macrophages were morphologically similar to immune cells identified using the pan-immune marker CD45, and were also found in similar locations after axotomy in wild-type mice (Figure 2). Macrophages were sporadically present within (Figure 2A, inset) and often near taste buds and nerve fibers of nerve-sectioned wild-type mice. Few CD68+ macrophages were observed in *Il1r* KO mice (Figure 2B).

At day 2 (Figure 2C), macrophage density significantly increased in CT-axotomized compared to sham wild-type mice (*p* = 0.0423). There were also significant main effects of mouse strain (*p* = 0.0423), and the interaction between strain and surgical treatment (*p* = 0.0015) (Table 1). Post-tests showed that increased CD68+ macrophage responses to injury in wild-type mice (*p* = 0.0013) were absent in *Il1r* KO mice (*p* = 0.0013). In conjunction with the CD45 analyses, these results demonstrate that early macrophage responses to taste bud denervation are suppressed in the absence of IL-1R signaling.

### 2.3. Immune Responses to Axotomy Are Primarily Composed of M2-like Macrophages

**Figure 3:**
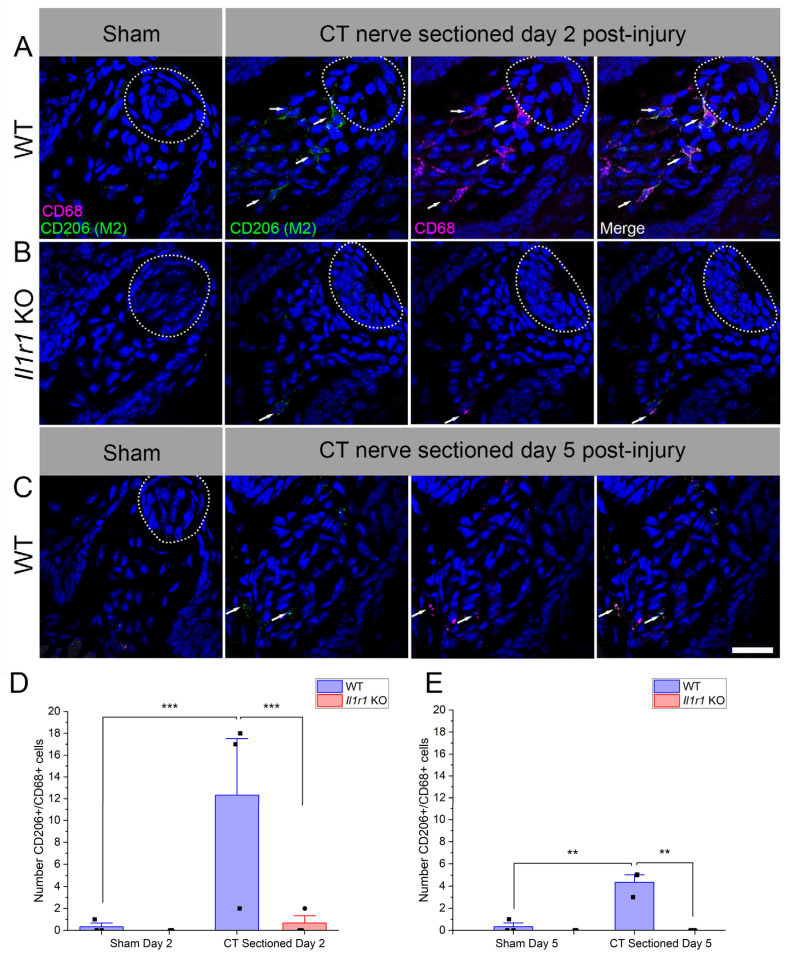
CD68+CD206+ M2-like macrophage responses to nerve injury predominate in wild-type mice. In these analyses, we counted double-positive macrophages, associated with a DAPI+ nucleus (blue), rather than quantifying immunopositive pixels as in Figure 1 and Figure 2. (**A**) Taste tissue, including fungiform papillae, taste buds (dotted white lines) and lamina propria, was populated by few macrophages in sham-sectioned wild-type mice. Double-positive macrophages were prominent in these regions in wild-type mice at day 2 after axotomy (white arrows). (**B**) At the same time, few macrophages were present in sham- or nerve-sectioned *Il1r* KO mice. (**C**) CD68+ CD206+ macrophages subsided by day 5 post-injury in wild-type mice, but remained elevated particularly at the base of papillae and in the lamina propria. The mean (+SEM) number of M2-like macrophages was significantly elevated in wild-type compared to sham-sectioned wild-type and axotomized *Il1r* KO mice at both (**D**) day 2 and (**E**) 5 (*n* =3 mice/group). ** *p* < 0.001; *** *p* < 0.0001. Scale bar in (**B**) = 20 µm.

Macrophages exhibit polarization into M1 and M2 subtypes, though little is known about their phenotype in normal or denervated taste papillae or the lingual epithelium. We refer to these macrophages as “M2-like” and “M1-like” (abbreviated as M2 and M1, respectively). Since macrophage responses were only elevated in the wild-type mice in which taste buds later regenerated, we tested whether M2 responses to axotomy are prevalent in this group. We used CD206 and CD68 to identify M2, double-positive macrophages (denoted as CD206++) in taste papillae and lamina propria based on findings in the tongue and other injured tissues [37,38,39,40]. Taste buds were not labeled with NTPdase2 in these experiments because of antibody compatibility, but were easily identified morphologically by DAPI (Figure 3A,B; white dotted lines). In sham-sectioned wild-type or *Il1r* KO mice, few macrophages were present in fungiform papillae and lamina propriae (Figure 3A,B).

At day 2 after axotomy, CD206++ macrophages were numerous in wild-type mice but sparse in *Il1r* KO animals (Figure 3A,B). A majority of CD68+ macrophages (56.6%) expressed the M2 marker CD206. There were significant effects of surgical treatment (*p* = 0.0002), mouse strain (*p* = 0.0003), and their interaction (*p* = 0.0005) (Figure 3D). Double-positive macrophages were significantly elevated in nerve-injured vs. sham-sectioned wild-type mice (*p* = 0.0001). In contrast, there was a significant difference between strains in the nerve-injured groups (*p* = 0.0001). Specifically, CD206++ macrophages remained at the baseline sham levels in *Il1r* KO mice.

Since CD206++ macrophage responses to injury were minimal at day 2, we also analyzed this population at day 5 to check for delayed responses in *Il1r* KO mice. M2-like macrophage responses followed a similar pattern at day 5 post-injury, although at lower levels in wild-type mice compared to day 2. However, there were significant effects of surgical treatment (*p* = 0.0044), mouse strain (*p* = 0.0018), and their interaction (*p* = 0.0044) (Table 1). As at day 2, responses were elevated in the wild-type group after axotomy (*p* = 0.002), and significantly reduced in *Il1r* KO vs. wild-type groups receiving CT nerve sectioning (*p* = 0.0013). In sum, M2-like macrophage responses were robust in control-strain mice, particularly at day 2, but negligible in the system-wide absence of IL-1R signaling.

### 2.4. M1-like Macrophages Were Absent at Day 2 and 5 After CT Nerve Injury

**Figure A1:**
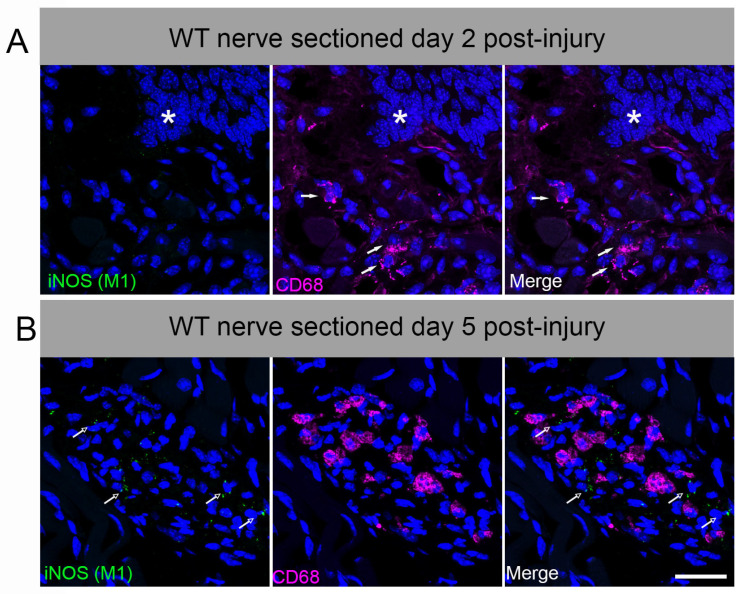
Lingual M1-like macrophages expressing iNOS are rare or absent after axotomy. Macrophages were identified with CD68 (magenta, arrows). (**A**) Macrophages in proximity to taste buds were increased at day 2 post-injury, as in Figure 2. However, iNOS was not co-expressed with CD68 in taste papillae or lamina propria. White asterisk marks the base of a fungiform papilla. (**B**) At day 5 post-axotomy, CD68+ macrophages are most prominent in the deep lingual muscle. Punctate iNOS was observed nearby, but rarely co-localized with macrophages (green, open arrows). DAPI (blue) was used to identify cell nuclei Scale bar in (**B**) = 25 µm.

We tested whether a subset of lingual CD68+ macrophages express the pro-inflammatory M1-like marker, inducible nitric oxide synthase (iNOS) [41]. We observed very limited iNOS expression in CD68+ macrophages deep in the lingual muscle and in the ventral glands at day 2 or 5 after CT sectioning in wild-type mice (Figure A1). Macrophages in taste papillae and nearby lingual mucosa remained iNOS-negative, though positive control staining was observed in lipopolsaccharide (LPS)-treated tongue and inflamed colon.

### 2.5. Taste Buds Degenerate in Both Wild-Type and Il1r KO Mice

**Figure 4:**
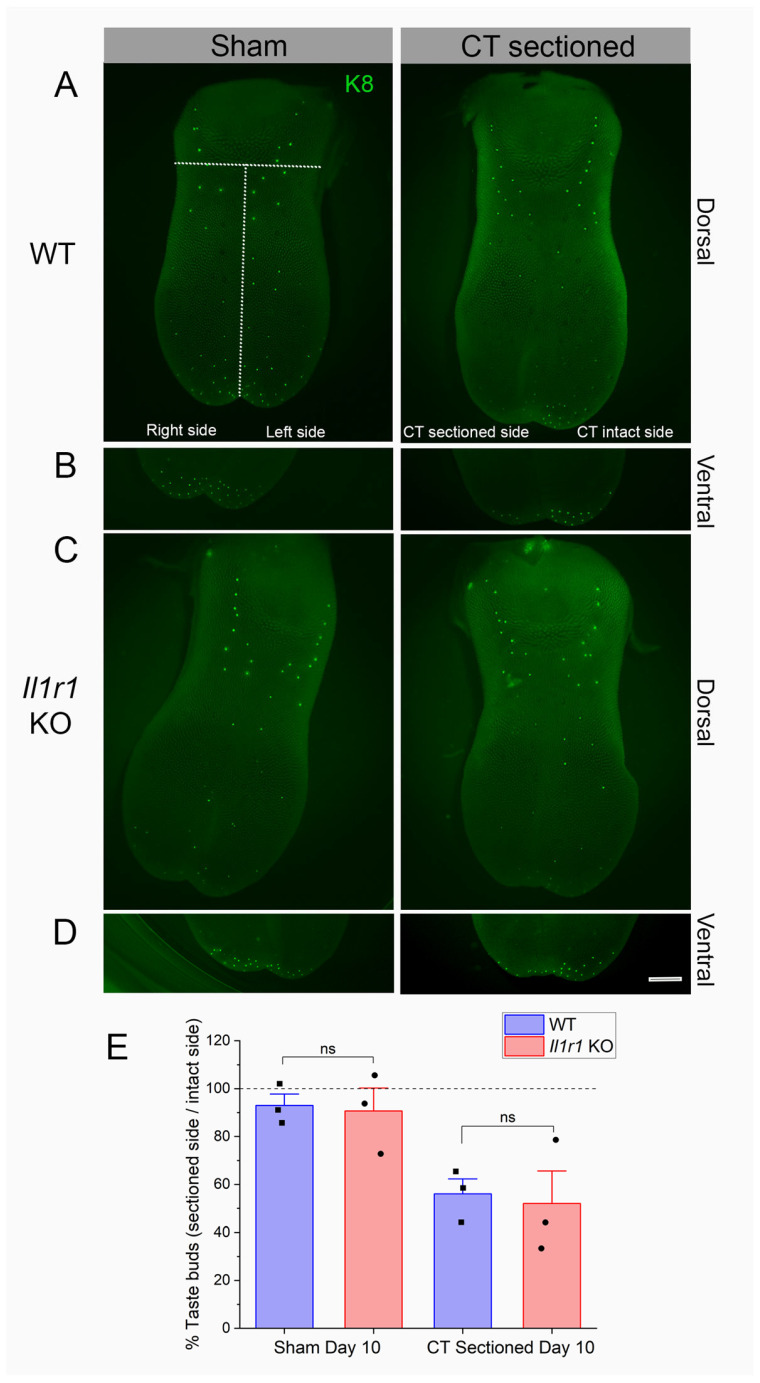
Taste buds degenerate in both wild-type and *Il1r* KO mice at day 10 post-injury. Lingual epithelia were enzymatically and manually dissected and taste buds visualized with Keratin 8 (K8). Taste buds are shown in sham-sectioned (**left**) or CT nerve-injured (**right**) lingual epithelia. Dotted lines in (**A**), upper left, show regions in which K8+ taste buds were counted anterior to the intermolar eminence, and on the right (sectioned) and left (intact) sides of the tongue. We counted taste buds on the (**A**,**C**) dorsal epithelium and (**B**,**D**) ventral side of the tongue tip, which has a high density of taste buds. Note the reduced density of green taste buds on the right (axotomized) dorsal and ventral tongues of both strains. (**E**) K8+ taste buds on the right side were expressed relative to the left (intact) side to calculate the percentage of degeneration. A percentage of 100% (horizontal dotted line) represents equal numbers of taste buds on the two sides of the tongue, or no degeneration. There were no significant differences between groups (ns), as detailed in the text. *n* = 3 mice/group. Scale bar in (**D**) = 1 mm.

We hypothesized that reduced immune responses to injury in *Il1r* KO papillae (Figure 1, Figure 2 and Figure 3) could further delay taste bud degeneration as a substrate for later deficits in regeneration and recovery in this strain. To test this, we counted taste buds expressing the marker Keratin (K)8 on the denervated and intact sides of lingual epithelial whole mounts at day 10 post-injury as shown in Figure 4A–D. As shown in Figure 4E, taste bud degeneration was similar in wild-type and *Il1r* KO mice. There was a significant main effect of surgical treatment (*p* = 0.0034) but not strain (*p* = 0.7415) (Table 1). There were no significant differences in post-tests, although sham vs. sectioned values approached significance for both wild-type (*p* = 0.0877) and *Il1r* KO (*p* = 0.0718) strains. Thus, taste buds degenerate at the same rate in the absence of IL-1R signaling despite suppressed macrophage responses to injury in these mice.

**Table 1:** Summary table for two-way ANOVA comparisons for major experiments.

Two-Way ANOVA Comparison		Main Factor	*F*-Statistic	df	*p*
Figure 1. CD45+ immune response	Day 2	Surgical treatment	20.83	1, 12	0.0006 ***
		Mouse strain	7.326		0.0191 *
		Interaction	20.87		0.0006 ***
	Day 5	Surgical treatment	0.7301	1, 8	0.4177
		Mouse strain	5.484		0.0473 *
		Interaction	4.102		0.0074
	Day 60	Surgical treatment	8.185	1, 9	0.0188 *
		Mouse strain	1.575		0.2411
		Interaction	0.4634		0.5132
Figure 2. CD68+ macrophage density	Day 2	Surgical treatment	5.068	1, 13	0.0423 *
		Mouse strain	5.068		0.0423 *
		Interaction	16.16		0.0015 **
Figure 3. M2-like macrophage density	Day 2	Surgical treatment	40.30	1, 8	0.0002 ***
		Mouse strain	36.09		0.0003 ***
		Interaction	32.23		0.0005 ***
	Day 5	Surgical treatment	15.44	1, 8	0.0044 **
		Mouse strain	20.96		0.0018 **
		Interaction	15.44		0.0044 **
Figure 4. Taste bud degeneration	Day 10	Surgical treatment	16.85	1, 8	0.0034 **
		Mouse strain	0.1167		0.7415
		Interaction	0.0084		0.9288

* *p* < 0.05. ** *p* < 0.01. *** *p* < 0.001.

## 3. Discussion

Immune responses to peripheral nerve axotomy and the consequences for subsequent axonal regeneration have been studied in depth [20,42,43,44]. Sterile inflammation in denervated target tissue, in contrast, is not well-understood, particularly in the taste system [6]. We previously demonstrated that IL-1R is required for normal taste bud regeneration and the restoration of gustatory function [7]. In the absence of signaling through this pleiotropic cytokine pathway, taste loss persists after experimental axotomy. Here, we report that leukocytes rapidly respond to CT nerve sectioning in control-strain mice that later recover normal taste function. The immune response is dominated by CD206+ M2-like macrophages which infiltrate the denervated anterior taste tissue within days. At the same early post-injury period (i.e., days 2 and 5), CD45+ leukocytes, CD68+ macrophages, and CD206++ M2 macrophages remain at sham-like control levels in the taste tissue of axotomized *Il1r* KO mice. Even 8 weeks after nerve sectioning, immune responses remain minimal in KO mice, indicating their absence rather than a delay.

While macrophage responses are more complex in vivo than in vitro [45,46], further phenotyping has provided insights into immune responses to axotomy and sterile tissue injury [43,47]. Similarly, this provides new information regarding immune responses to axotomy, from which wild-type mice successfully recover taste function. M2 macrophages are associated with anti-inflammatory responses and tissue repair [41,45,48,49]. iNOS is not constitutively expressed, but upregulated by inflammatory stimuli, including cytokines, LPS, and peripheral nerve injury [50]. Systemic LPS also induced iNOS in circumvallate taste buds and nerve fibers in previous reports [51].

We previously demonstrated similar numbers of taste buds in wild-type and *Il1r* KO mice at day 5 following CT nerve axotomy [7]. Here, we tested whether suppressed immune responses further delay taste receptor cell degeneration in *Il1r* KO mice, but that is not the case. Instead, taste bud numbers are similar at day 5 [7] and 10 post-injury across mouse strains, despite the absence of CD45+ leukocyte responses in KO mice. Neurophysiological responses to taste stimuli return at day 11-17 after CT axotomy in a subset of wild-type mice, indicating that nerve fibers and new taste receptor cells are reconnecting at this period. Partial regeneration at this period likely explains why the reduced number of taste buds in axotomized vs. sham mice approaches but does not reach significance. It is unlikely that *Il1r* KO delays taste bud degeneration beyond day 10, since the CT nerve remains unresponsive to taste stimuli from day 11-60 post-injury in these mice. Our prediction was based on the likelihood that CD45+ immune cells, and particularly macrophages, clear apoptotic taste receptor cells. CD45 is expressed on all cells of hematopoietic lineage, including lymphocytes (e.g., T cells and B cells), innate immune cells, dendritic cells, and at least some fibroblasts.

Our finding that taste bud degeneration proceeds in the absence of infiltrating leukocytes highlights the uncertainty over the fate of denervated, apoptotic taste receptor cells [52]. Suzuki and colleagues reported that macrophages phagocytose nerve fibers, while fibroblasts engulf taste receptor cells in posterior mouse taste buds after glossopharyngeal nerve sectioning [18]. This may be consistent with our results if CD45-negative fibroblasts phagocytose anterior taste receptor cells while M2-like macrophages clear taste nerve fiber debris, though advanced imaging techniques such as intravital and light-sheet microscopy of the anterior taste field would be required to closely study these proposed interactions. A small subpopulation of CD45+ cells isolated from murine tongue comprised of fibroblasts in a recent study using single-cell RNA sequencing [53]. However, the sizable population of vimentin-positive stromal cells in other reports suggests that many fibroblasts are CD45-negative [54,55]. Fibroblasts play a role in wound-healing in the tongue, and are suggested to recruit macrophages to the injury site [56]. Taste cells may also be cleared by type I, glial-like taste cells [57,58]. Desquamation through the taste pore has been suggested as a mechanism for taste receptor cell clearance, though it is unclear whether this mechanism could accommodate widescale taste cell death after axotomy [4,5].

Two novel, major subpopulations of CD45+ macrophages in uninjured mouse tongue were recently reported. Cx3cr1+ macrophages occupy the taste papillae and lamina propria, while Folr2+ cells reside in the lamina propria and deeper lingual muscle [53]. Both of these resident macrophage populations express CD68 [53]. The spatial segregation (i.e., taste papillae and lamina propria vs. muscle) and morphological differences in the two CD68+ macrophage subsets in the current study may correspond to these populations, though additional immune responses are likely induced by nerve sectioning. The authors suggest that Cx3Cr1+ macrophages may communicate with nearby nerve fibers during homeostasis and after injury, as was recently reported in skin [59]. Thus, even though taste buds degenerate at the same rate in wild-type and *Il1r* KO mice, we cannot rule out interactions between macrophages in papillae, lamina propria and muscle with CT nerve fibers. Since anterior taste buds are dependent on trophic support from the CT nerve, major changes in the rate that gustatory fibers degenerate are unlikely [6]. In a previous study, we also found that the subset of taste buds that regenerate (~40%) in *Il1r* KO mice are innervated. In fact, the percent of the taste buds occupied by CT fibers is slightly elevated in the absence of IL-1R signaling at day 56-60 post-injury [7].

IL-1 is a master regulatory cytokine. Knocking out its receptor will inhibit many inflammatory signals that stimulate immune responses to axotomy and subsequent nerve and taste receptor cell degeneration [60,61]. While resident CD45+ populations are similar during homeostasis (e.g., sham wild-type vs. KO groups), injury unmasks the critical role of IL-1R cytokine signaling in taste regeneration and recovery [7]. For example, macrophage chemoattractant protein (MCP)-1, intracellular adhesion molecule (ICAM)-1 and vascular adhesion molecule (VCAM)-1 are upregulated at 24 and 48 h after CT nerve axotomy in rats [26,27]. IL-1 signaling is a major inducer of these recruitment signals, which likely results in the suppressed immune responses to nerve injury reported here [60,61,62]. One of the challenges in identifying the molecular mechanisms by which IL-1 signaling contributes to taste regeneration is the huge number of biological processes that it controls [60,61,62]. The current study is a step in that direction, since we report that M2-like macrophages respond to CT sectioning in control but not *Il1r* KO mice. Importantly, taste buds degenerate at the same rate in both strains. Even though this is a negative result, it shifts the focus of future studies to later regenerative processes.

The differences observed in taste bud regeneration and functional recovery between WT and *Il1r* KO mice after CT nerve axotomy may reveal a potential growth factor-like role for IL-1 signaling in the injured taste system. We hypothesize that this cytokine could target taste progenitor cell proliferation, differentiation, or their ability to attract regenerating CT nerve fibers. One example of a similar non-canonical role for IL-1 occurs in skin wound healing. Keratinocyte-derived IL-1 stimulates growth factor release, including keratinocyte growth factor (KGF)-1 and fibroblast growth factor (FGF)-7 from fibroblasts to help regulate skin wound repair [63]. In the gut, IL-1 and other cytokines induce intestinal stem cell proliferation fostering regeneration and repair [64]. The olfactory epithelium also upregulates *Il1b* and other pro-inflammatory cytokines in a tumor necrosis factor (*Tnf*)-overexpression model of chronic inflammation. Horizontal basal stem cells recruit macrophages and other leukocytes through an NFkB-dependent mechanism that is acutely pro-regenerative but chronically detrimental to olfactory sensory neuron regeneration and functional recovery [65,66]. The requirement for tightly regulated inflammatory responses might be similar for taste bud regeneration, but this remains to be tested.

We have shown differential changes in immune cell populations in the anterior lingual taste field after CT nerve axotomy in wild-type and *Il1r* KO mice. However, a limitation of this study includes a reliance on morphology and image analyses. While this technical approach allowed us to focus on immune cells in close proximity to denervated taste buds, further studies are needed to understand how these identified immune cell populations interact with taste cells and nerve fibers and functional consequences. Our results suggest that the M2 macrophage population is associated with taste bud regeneration, though further conclusive work is needed after axotomy and certainly in other models of taste loss. Importantly, better understanding of neuroimmune mechanisms leading to taste recovery could suggest new strategies to treat the significant clinical problem of taste dysfunction from chemotherapy, injury, and long COVID-19 [67,68].

## 4. Materials and Methods

### 4.1. Animals

The adult male and female mice were 8–16 weeks old during all of the experimental procedures. Wild-type C57BL/6J (WT) and B6.129S7-Il1r1^tm1lmx^/J mice were obtained from the Jackson Laboratory (000664; #003245, respectively, Bar Harbor, ME, USA) and bred in-house. The mice were chosen at random from at least two different litters per group. Sex differences were not observed in previous studies focused on CT sectioning in rodents [7,19,23,24,25,27]. The genotypes were confirmed using Jackson Laboratory protocols on-site or by outsourcing (Transnetyx, Cordova, TN, USA). Specified pathogen-free mice were kept on a 12:12 h light:dark cycle with ad libitum access to rodent chow (Envigo Teklad, Madison, WI, USA) and filtered tap water. This study followed ARRIVE guidelines https://arriveguidelines.org/arrive-guidelines (accessed on 29 December 2025).

### 4.2. Chorda Tympani (CT) Nerve Sectioning

A ketamine (50 mg/kg) and xylazine (10 mg/kg) cocktail was administered intraperitoneally to anesthetize mice. Once a surgical plane of anesthesia was achieved, mice were moved to a water-circulating heating pad to maintain body temperature. The mice were given a ketoprofen analgesic (5 mg/kg bw) prior to the start of surgery. CT nerve sectioning was done as previously described [7]. Access to the CT nerve was gained ventrally through the neck and transected after bifurcation from the lingual nerve, with the severed ends being left in place. The sham-sectioned mice underwent the same procedure; however, the CT nerve remained intact.

### 4.3. Tissue Collection and Immunofluorescence

Two, five, or 60 days after CT nerve sectioning, mice were sacrificed, and the tongues were removed with the mandible still attached. Samples were fixed in paraformaldehyde for 5–7 h then the tongues dissected free and placed in cryoprotectant overnight [7,69]. Tongues were frozen in O.C.T (Fisher, Hampton, NH, USA) and 8 µm sagittal sections were obtained for immunostaining. Samples were rinsed with PBS (7.5 pH) and placed in a 5% blocking solution for 45 min for CD45 analysis and 30 min for CD68 and CD206 at room temperature. Staining for iNOS followed a similar procedure as described; however, we included a 30 min permeabilization step in 0.5% triton-x in PBS before placing samples in the blocking solution (Sigma-Aldrich 9036-19-5, St. Louis, MO, USA). NTPdase2 (1:1000, Labome; Lambertville, NJ, USA) was used to stain Type I taste cells to identify the taste buds. We used NTPdase2 instead of Keratin 8 since antigen retrieval interfered with immune cell staining. CD45 (1:100; R&D Systems; Minneapolis, MN, USA) is a pan-immune cell marker, while CD68 (1:100; Abcam; Cambridge, UK) identifies macrophages. To observe the M1 and M2 macrophage subtypes, iNOS (1:100; Abcam; Cambridge, UK) and CD206 (1:1000; Abcam; Cambridge, UK) were used, respectively. Tongue sections were incubated in primary antibodies in a humidified box at 4 °C overnight. Samples were rinsed with PBS and incubated in donkey anti-goat 594 (1:500; Abcam; Cambridge, UK), donkey anti-rabbit 488 (1:1000; Jackson ImmunoResearch; West Grove, PA, USA), goat anti-rabbit 488 (1:1000; Jackson ImmunoResearch; West Grove, PA, USA) and goat anti-rat 594 (1:500; Abcam; Cambridge, UK) in a humidified box at room temperature for 1 hr. uclei were stained with DAPI(#D1306; Invitrogen/Fisher Scientific, Hampton, NH, USA) and slides coverslipped with Fluoromount-G (#0100-01: Southern Biotechnology, Birmingham, AL, USA).

### 4.4. Whole-Mount Lingual Epithelium Analysis

*Il1r* KO or wild-type mice received CT nerve axotomy and were then euthanized with isofluorane followed by bilateral thoracotomy 10 days later and the whole tongues were dissected from the jaw. We selected this period since we previously found no difference in the number of taste buds remaining at day 5 post-injury in wild-type or *Il1r* KO mice [7]. The tongues were washed in PBS and injected with ~1.5 mL collagenase A (1 mg/mL, catalog no. 10103578001; Roche, Basel, Switzerlnd) and dispase II (2.5 mg/mL, D4693-1G; Sigma, St. Louis, MO, USA). After injection, the tongues were incubated in 300mL of PBS with an oxygen bubbler for 45 min at room temperature, then placed on a shaker and fixed in 4% PFA for 1 h. Intact whole epithelia were dissected from the underlying muscle and washed in PBS. Lingual epithelia were placed in blocking solution for 1 h then incubated in the primary antibody K8 TROMA (1:500; DSHB, Iowa City, IA, USA) for 2 days to identify the taste buds. epithelia were washed with PBS, incubated in secondary antibody donkey anti-rat 488 (1:500, Jackson ImmunoResearch; West Grove, PA, USA) for 2 days, and counterstained with DAPI in ddH_2_O. Specific antibody information can be found in Table A1.

We counted taste buds on the right/denervated or left/intact side of the tongue (in sham- or chorda tympani nerve-sectioned mice) on images taken with a Leica M165 FC stereoscope. Separate images were taken of the dorsal epithelium and the ventral tip which has a high density of fungiform taste buds. We expressed the number of taste buds on the right or injured side of the tongue relative to the number on the intact side to obtain the percent degeneration (number of taste buds on right or sectioned side/number on the left or intact side × 100). Brightness and contrast were adjusted to assist in counting the taste buds.

### 4.5. Image Analysis

Non-overlapping images of either taste buds, papilla or in the absence of either, lamina propria, were taken at 40x magnification. CD45^+^ and CD68^+^ pixels were thresholded and calculated as % thresholded area relative to the region of interest (ROI 581.5065 mm^2^) [7,19,24,70]. The experimenter was blinded to surgical treatment and strain for most analyses. A random number generator was used to assign slides a number, slides were then ordered accordingly and slide labels were covered. The analyzer was not involved in the blinding process. Serial sagittal sections were analyzed using a BX51 microscope with epifluorescence (Evident Scientific, Waltham, MA, USA), a digital monochrome camera (Cool Snap, Roper Scientific, Trenton, NJ, USA), and MetaMorph software 7.5.6.0 (MDS Analytical Technologies, Sunnyvale, CA, USA). Quantification of iNOS- and CD206-positive cells was done using the same epifluorescence microscope as previously described. Individual cells were considered double-positive for CD68 and iNOS or CD206 if colocalization was found around DAPI-positive nuclei and could be distinguished from the background. For presentation purposes, z-stack images with a step size of 1 µm were also captured with a Nikon A1R confocal/multiphoton microscope. Images of the taste buds were captured at a magnification of 60× with a 3× zoom setting. We then converted images from NIS Elements (version 4.30.01) to maximum Z projections using ImageJ (version 27.3). Images for Figure A1 were captured with a Leica Stellaris confocal system at 40× (2.5× zoom) and a 1 µm step size, and LAX .lif files converted to maximum Z projections in ImageJ. Images were minimally adjusted for brightness and contrast in Adobe Photoshop with the same settings applied equally to all panels.

### 4.6. Statistical Analysis

Two-way ANOVAs were performed in GraphPad Prism (version 10.6.0), using mouse strain (wild-type or *Il1r* KO) and surgical treatment (sham- or CT-sectioned) as the main factors. Bonferroni post-tests were performed on selected comparisons: (1) sham-sectioned wild-type vs. *Il1r* KO mice; (2) CT-sectioned wild-type vs. *Il1r* KO mice to detect strain-related differences in the baseline and injury-induced immune responses, respectively; and (3) sham- vs. CT-sectioned groups within each strain to detect the effects of surgical treatment. Bonferroni post-tests were used for multiple comparisons. *p* values ≤ 0.05 were considered significant. A summary of the *F* values, degrees of freedom, and *p* values are reported in Table 1.

## 5. Conclusions

Currently it is unclear how specific innate immune cell populations are dynamically affected by CT nerve axotomy in wild-type mice or in the absence of IL1-R signaling. Our objective was to quantify immune responses (CD45+ leukocytes, M1 and M2 macrophage subtypes) in both mouse strains after nerve transection. We report significantly increased M2-like, pro-regenerative macrophages near denervated taste buds in WT but not in *Il1r* KO mice at day 2 and 5 post-injury. However, similar numbers of taste buds degenerated by day 10 post-axotomy in both mouse strains. Long-term immune response dysregulation was not observed in *Il1r* KO mice in which most taste buds fail to regenerate leading to functional deficits. These results suggest that additional injury-induced mechanisms may influence taste bud degeneration, regeneration, and the recovery of taste responsivity. However, the finding that M2-like macrophage responses predominate in wild-type mice, which soon recover normal neural taste function, may provide a novel avenue to improve taste bud regeneration after injury, infection, or cancer treatment.

## Data Availability

The original contributions presented in this study are included in the article. Further inquiries can be directed to the corresponding author.

## Appendix A

**Table A1:** Primary and secondary antibodies used in study.

Primary Antibodies	Catalog # and Source	Reactivity	Concentration	Secondary Antibodies
NTPdase2	501943785; Labome	Rabbit anti-mouse	1:1000	Donkey anti-rabbit 488; 1:1000
CD45	AF114; R&D Systems	Goat anti-mouse	1:100	Donkey anti-goat 594; 1:500
CD68	Ab53444; Abcam	Rat anti-mouse	1:100	Goat anti-rat 594; 1:500
iNOS	Ab3523; Abcam	Rabbit anti-mouse	1:100	Goat anti-rabbit 488; 1:1000
CD206	Ab64693; Abcam	Rabbit anti-mouse	1:1000	Goat anti-rabbit 488; 1:1000
K8 TROMA	DSHB	Rat anti-mouse	1:500	Donkey anti-rat 488; 1:500
